# Practical Medicine, Materia Medica and Physiology

**Published:** 1877-07

**Authors:** Wm. J. Maynard


### REPORTS OF THE SECTIONS.

### SECTION I. PRACTICAL MEDICINE, MATERIA MEDICA AND PHYSIOLOGY.

(Reported by Wm. J. Maynard, A., M. M. D.)

First Day, June 5th.

Treatment of Croupous Pneumonia. By A. B. Palmer, of Michigan.—In the absence of Dr. Palmer the paper was read by Dr. N. S. Davis. He recognized three forms of this disease, croupous, catarrhal and interstitial. He regarded it as a specific inflammation, a peculiar exudation and a self limited disease. The percentage was 6 1-10 of all diseases, and by far the largest number ran a mild course and recovered in about 10 days, but he thought the lungs never fully recovered their original healthy condition. He said there was no lack of discussion among those advocating the heroic, stimulant and expectant treatments. He regarded the heroic plan of managing these cases, especially among the poor, as worse than useless. He had formerly thought that opiates would cut short the disease in the outset quicker than anything else, and always used them; after their effect, giving a few grains of blue mass, followed by a saline cathartic, and then the eliminating treatment by acetate of potash, etc. He used to resort also to bleeding in plethoric subjects, but this would never cut short the disease. Since 1865, he had used quinine in every case treated, and with great effect; had seen many cases with no malarial poisoning, and with full symptoms of the disease, cut short very speedily by full doses of this drug. He agreed with Juergensen, that "pneumonia was a general and specific disease, not dependent upon a local cause, but belonged to the infectious diseases, that nature cured, and all that was necessary for the physician to do, was to sustain the patient and to prevent the failure of the heart's action, which the fever had a tendency to do." J. relied upon the cold bath and large doses of quinine in his treatment. He took issue with Juergensen that the disease always ran a regular course, but thought that he had been able to abort it in many instances by large doses of quinine, and the reports of others had shown the same results. He thought the cases shown by J., were mostly in hospital and in consultation, and therefore seen too late to abort. He contended that pneumonia was not an obstinate type of disease; that opium would sometimes arrest it early in the attack; so would alcohol or veratrum viride. These agents, however, would depress the heart's action secondarily, and this must be guarded against. Quinia does not have this action, but more speedily relieves internal congestion, induces perspiration, diminishes exudation, and acts through the nervous system. A good deal depended upon the time of administration. In 12 to 24 hours after the chill, he gave 6 to 10 grs. of quinia with $\frac{1}{8}$ of a grain of morphia, afterwards 4 to 8 grs. every 2 to 3, hours with smaller doses of morphia, until 50 or 60 grains of the quinine had been taken. Sometimes 25 grains would be sufficient, with this came a marked diminution in the pulse, a fall of temperature, slower respiration, in short the disease was aborted. All that would be necessary after this was a laxative of blue mass, followed by a saline cathartic and elimination by the kidneys. If the disease was seen later in its course, the morphia should be given in smaller quantities but the quinia in the same doses.

In the discussion of the paper, Dr. Gallagher thought the disease would get well without much treatment under good hygienic rules and good diet, and that the disease was much milder than it was 30 years ago; then the fever and pain being more intense required the lancet; but at the present time he regarded it improper. He doubted very much the power of quinine to control the disease when there was no malarial complication. He depended upon aconite and good diet.

Dr. Hibbard, of Indiana, always looked for the natural tendency of the disease, and was careful about using remedies that would do any harm.

Dr. Scott, of Ohio, said, if his pathology was correct, he could assist very much the obstruction to the flow of blood. In his opinion, the teachings of Todd and Ziemsen had done a great deal of harm, and the lancet and calomel to-day were as potent as any remedies at our command. He did not believe that the lungs never recovered from an attack of this kind, but thought that they fully recovered their functions, even if exudation was never thoroughly absorbed.

Dr. Schenck, of Kansas, differed from his Indiana friend (Hibbard), and observed that a great amount of good could be done by the physician. He liked the supporting plan of treatment better than anything he had ever tried, and was inclined to agree with Dr. Palmer in the assertion that quinia was a very powerful agent in controlling the disease, as he had once an occasion to try it on his own person when suffering from such an attack.

Dr. Murphy, of Cincinnati, did not believe that quinia, nor any other drug would be able to abort pneumonia. The very best authorities agreed that the disease would run a natural course and was self limited. In his opinion there was not much of value in the paper anyway. He thought the main thing for the practitioner to do, was to look out for complication, the pneumonia would get well of itself.

Dr. Yandell, of Kentucky, did not believe pneumonia a disease of itself, but rather the result of malaria or a catarrhal condition. The cause should always be found out and treated, whether this be malaria, struma or syphilis. He had seen malaria everywhere, and did not believe there was a place known where it might not abound. It was the cause of most acute diseases.

Dr. Carpenter, of Iowa, thought that in this disease the patient should be well supported from the start.

Dr. Bailey, of Louisville, thought the disease was the same now as it always had been, and always resorted to antiphlogistic remedies. He thought the cold bath, as had been suggested, very injurious, but believed he had seen the very best results from quinine.

The Effects of Medicine in Small Doses. By Dr. John Morris, of Maryland.—The writer thought that altogether too much medicine was given by the profession; that a great many drugs were used in such quantities as could only in part become absorbed; that they then acted as irritants, or passed away without doing any good. He spoke at length of the following drugs, the peculiar action of each when used in minute doses, and the diseases in which they were particularly valuable, *i. e.*, Calomel, Iron, Quinine, Alcohol, Tart, Antimony, Ipecac, Aloes, Rhubarb, Opium, Gelseminum, Squills and Ergot. Nothing new was elicited from this resume of materia medica, and the paper closed with the following deductions:

1st. That the true physiological effect of medicine may be best attained by the administration of small doses frequently repeated.

2d. That medicines thus given are cumulative in their operation.

3d. That the effect of remedies is greatly increased by combination, the manner of preparation, the time and mode of administration.

4th. That large doses of remedies act as irritants; that they produce an abnormal state of the blood, as is evidenced by such conditions as narcotism, alcoholism, iodism, ergotism and bromidism.

5th. That more especial attention should be given at the bed side to the influence of remedial agents, to the end that a greater certainty may be exercised in their prescription.

### Second Day, June 6.

Report on Clinical and Meteorological Records. By Dr. N. S. Davis, Chicago.—After speaking of the importance of the work under consideration, as the only true method by which we may be able to get at the cause of disease, he hoped for a more extended co-operation on the part of medical men in different parts of the country. For some time past he had been making close observations in this city on the subject. He produced statistics and abundant evidence from meteorological reports, not only here but in Cairo, Davenport and Omaha, to show that the meteorological changes in these parts, giving a high range of temperature and humid atmosphere, co-existed with certain body changes.

The conclusions of the paper were:

1st. That bowel affections begin their annual prevalence when continuous warm weather prevails.

2d. That every subsequent occurrence of several days and nights of high temperature, causes new attacks to be incurred throughout the month of July, less in August, and still less in September.

3d. That it is not simply extreme heat, but the continuance of it for several days.

4th. That continuous high heat, to be effective, must follow a protracted season of cold.

5th. If we compare these deductions with mortality, we find that they are confirmed.

Cases Showing the Influence of Colorado Climate in Consumption. By Dr. Chas. Dennison, Colorado.—This paper was a supplement to a report made by him last year. He said he wished to avoid the criticism of a certain lengthy report of doubtful cases, "that its publication was very unfortunate for the writer, as well as for the invalids;" that he preferred to look mainly on the bright side, to run the risk of his conclusions being called rose colored by the inconsiderate reader. At the same time he felt keenly the responsibility of drawing conclusions which would lead those seriously diseased to try to prolong their lives in the Rocky Mountain region. The pros and cons should be equally and faithfully represented till at length we could decide, by the evidence of past experience, the best course for all forms of phthisical patients to pursue, so far as climate is concerned. He then gave the history of six favorable and six unfavorable cases, that were chosen from the records of over 100 phthisical patients that had been under his care. From the analysis of these he concluded, that the favorable influence of high altitude in phthisis is best shown in the incipiency of chronic inflammatory and hæmorrhagic cases, and in others as these characteristics exist, the measure and severity of disease, the inheritance and mental state being taken into account, and a partial recovery necessitating a permanent residence.

On the other hand, the unfavorable influence of high altitude on phthisis is shown as the disease approaches or is complicated with the following conditions: Cardiac disease associated with increased labor and abnormal activity, the stage of softening in acute cases (especially with a uniformly rapid pulse and high temperature) associated with extensive deposit, irritable nervous state, and lack of courage to do in order to be well.

Dr. Bowditch, of Boston, referred to cases where the lower part of the lungs were first affected, and the patients visited California with considerable benefit, and finally recovered entirely after a residence of a year or more in Europe. He had observed also that patients would go to Colorado and other places and get better, and the moment they returned to their old locality they would get worse again. He had always been doubtful about sending patients away from home when they had reached the stage where crackling sounds could be heard in the lungs. He was of the opinion that if patients were sent to Colorado, they should remain there permanently, if they received any benefit from such a change of climate.

Dr. Ulrich, of Pa., remarked that there was a general agreement in the profession, that the object of treatment in phthisis was to better the general condition of the patient, and support nutrition by proper food, and surround him with circumstances favorable to the proper assimulation of that food. He was living in a manufacturing city, and among the mills were found many cases of phthisis, and it became his duty to prescribe for such cases very often. Whenever he was able to induce them to leave their in-door employment, and the impure atmosphere, for some more active, out-door occupation, and surround them with better hygiene, he found that the disease was very much ameliorated, and in some absolutely cured. He thought it was getting too fashionable to send patients away, when a radical change of occupation and living at home would be equally beneficial.

Dr. Kingsley referred to the fact, that patients going from St. Louis to Colorado were very much benefited, but upon returning, the disease had developed rapidly, and the patients died in a very short time.

Dr. Scott, of Ohio, was inclined to agree with Dr. Ulrich, that by change in occupation and mode of living at home, about as much good could be obtained as by sending patients away.

### THIRD DAY, JUNE 7.

REPORT OF COMMITTEE ON BOVINE VACCINATION. DR. H. A. MARTIN, MASS.—Dr. M. said this work was first introduced by him in 1870, and his is now the only original part of the Beaugency stock as it existed in Paris before the war. He presented in full the disadvantages of the other method when compared with the one under discussion. Regarding the arguments that have been offered against the system, *i. e.*, that the virus could not be kept fresh for any length of time, the greater liability to the production of erysipelas, herpes, etc., he declared that these results do not follow when proper care is exercised in selection and management. The claims in favor of this mode of vaccination were:

1st. Its unlimited supply. He had furnished 5,000 points a day, and could produce 50,000 if necessary.

2d. Its absolute immunity from the possibility of inoculating other diseases. To show that syphilis could be inoculated by vaccination, he related the fourteen cases that were reported by Hutchinson, of London.

At this point the speaker's time expired, and he was obliged to stop.

Dr. Scott, of Ohio, inquired of Dr. Martin whether he had any experience with vaccination with matter taken from a heifer that had been inoculated with small-pox. Dr. Martin replied that he had no experience of his own, nor should he ever have, because he was certain that small-pox could be transmitted in that manner.

Dr. Griffith inquired of Dr. Martin if he had any knowledge of any authentic case in which variola had occurred after the use of animal vaccination. Dr. Martin replied in the negative, and added his experience in confirmation of the statement that vaccination and re-vaccination rendered the person safe for life. He did not believe that a satisfactory re-vaccination was ever followed by small-pox.

On the Recognition and Management of the Gouty State in Diseases of the Skin. Dr. L. Duncan Bulkley, New York.—Dr. B. remarked that this peculiar condition of the system was frequently overlooked by practitioners in treating chronic skin diseases, and that the gouty state was not often treated unless there was actual deposit and pain in the joints. But if we would do the most good in these cases, we must go further back than the actual blood condition, and arrest the trouble in its earlier manifestations. These manifestations were generally disordered conditions of the stomach, a train of phenomena known as dyspepsia. There was usually flatulence and eructation after eating. Impairment of appetite, pale and flabby tongue, constipation, obstruction to the flow of bile, palpitation of the heart and dyspnœa, were common, also languor and inaptitude for exertion, drowsiness after meals, and waking from sleep in an irritable condition. The urine should be frequently and thoroughly examined in every case, and deductions made. In the management of these patients great care should be given to diet and hygiene. To keep up a proper action of the liver, and to relieve constipation, he relied upon blue mass and colocynth. In other cases he gave aloes and iron. For other primary dyspeptic symptoms he was very careful to select such drugs as would best meet the indications. He had found diuretics very valuable, and generally preferred acetate of potash in 30 grain doses, a few hours after meals. He had used also the salts of lithia and Kissingen water with much benefit.

Dr. Hibbard, of Indiana, thought there was great difficulty in specifying what kind of diet in any individual case was best adapted to the disorders of the digestive apparatus, and that we made a mistake if we laid down any definite rules with regard to it.

Dr. Cabell, of Virginia, inquired of Dr. Bulkley whether he had used any other mineral water except those he had mentioned. Dr. Bulkley replied he had used the German bitter waters, but had not been as well satisfied with their effects as with those produced by repeated doses of Kissingen. With reference to the effect produced by visiting springs, he thought it was the effect produced by the change of climate upon the gouty state, rather than any direct effect upon the skin disease, which accounted for the good results which sometimes followed a visit to those places, and the use of the water. Dr. Cabell thought that the thermal baths had a very good effect in these troubles. In using the German mineral waters, his experience had been different from Dr. Bulkley. He coincided entirely with Sir Henry Thompson in ascribing to them, when taken regularly in small quantities for months, a much more permanent effect than could be expected from the use of any of the antacids, such as lithia or any other alkali.

Dr. Duhring, of Philadelphia, said he wished to corroborate what Dr. Bulkley had said; he had, however, some difficulty in making out just what Dr. B. meant by the gouty state, but as that was a topic too broad to be entered upon at that time, he wished to ask, what diseases of the skin he included in that condition known as the gouty state? He presumed eczema, psoriasis, etc. Dr. Bulkley replied that he considered acne, furuncles, lichen, and urticaria as manifestations of the gouty state.

A Study of Nine Hundred and Sixty-five Cases of Chronic Pulmonary Diseases. Dy Dr. F. H. Davis.—This record showed that of this number, chronic catarrhal bronchitis was the most common, and 403 cases are recorded. Dr. D. thought that in chronic bronchitis, if the patient could avail himself of that expensive prescription, a change of climate, a cure could be obtained. In 15 cases where this could not be done, he had effected a cure by compressed and rarefied air inhalations. He had found that inhalation of compressed air, followed by exhalation into rarefied air, diminished the capillary congestion and consequent hypertrophy of the mucous lining of the bronchi, and restored the tone and contractility of these vessels. To be efficient it should be followed up for six to twelve months, and accompanied by a strict regulation of the habits of living, clothing, exercise, etc. In these cases the most efficient drug he had found, was the muriate of ammonia in combination with some anodyne and expectorant. The addition of six to eight drops of chloroform was very beneficial in combination with each dose to some otherwise intractable cases. Among this whole number there were but 30 who showed signs of any constitutional impairment, but the general vigor, appetite and digestion was up to the ordinary standard, proportional to the age.

This record contained 119 cases of chronic bronchitis, presenting a special gastric and nervous complication. In addition to the usual physical signs of chronic catarrhal bronchitis, we find in these cases symptoms of indigestion and gastric irritability; a lack of tone of the digestive organs, a tendency to acidity and fermentation following the ingestion of food. These cases occurred among those who had been addicted to the excessive use of alcohol and strong tea. In these cases treatment should be directed primarily towards controlling the gastric and nervous irritability. He had used with the most success, a combination of liq. ammoniae acetatis, syr. ipecac and tinct. opium. The œnothera biennis, (evening primrose) either in decoction or the fl. ext., would relieve promptly some of these cases where other means had failed.

There were recorded 283 cases as chronic rheumatic bronchitis. In these cases auscultation revealed sonorous and sibilant rales, rather than the mucous rales of the catarrhal variety, with harsh, dry cough and scanty expectoration. Associated with this was more or less evidence of general muscular and articular rheumatism of a chronic grade. In these patients colchicum acted almost as a specific, affording prompt and certain relief. The record contained 123 cases of phthisis pulmonalis; 56 of these were hereditary, and the remaining number were caused by apparently different agencies at work, *i. e.*, alcohol, constitutional syphilis, extreme poverty and consequent unhealthy living and surroundings, together with insufficient clothing, exposure, hard work, etc. He thought the proper essentials to success in the treatment of these cases, were proper food and clothing, good hygienic surroundings, regular habits of living, out-door exercise, but not with prolonged exposure to inclement weather, and a regular systematic practice of deep respirations. This could be best attained by the inhalation of compressed air, as before mentioned—an apparatus for which he fully described. The only other element of treatment was cod-liver oil, or ext. of malt, to build up the strength of the patient.

Dr. Scott, of Ohio, directed the attention of the section to the fact that Dr. Flint had published a monogram upon tuberculosis of the lungs, in which he said " that the deposit took place in the air vesicles." He did not believe that; he thought that any deposit which took place in consequence of arrest in the activity of the circulation of the lung, occurred outside of the air vesicles. Any condition of the atmosphere, or a poisoned condition of the blood, such as would interfere with the functions of the lungs, would cause an arrest of the circulation at that point, and not in the air vesicles.

Dr. Lester, of Missouri, said that Dr. Davis had stated that consolidation of the lung, resulting from bronchitis, occurred but rarely in infants. He believed that catarrhal inflammation of the bronchial mucous membrane was of frequent occurrence in infants, and that they died of collapse of the lung as a result, but as a chronic condition it was rare. He thought, also, that nine-tenths of all the cases of consumption were of inflammatory origin, following catarrhal pneumonia. He thought that tubercular phthisis was a rare disease in the western part of the country, especially in the mining districts.

Dr. Waterman, of Indiana, remarked that while he did not discard the use of local remedies in the treatment of consumption, the principal theory was, how to increase healthy blood, and an active circulation through the lungs. His plan was to stimulate the stomach with whisky, then give it good food and stimulate digestion by the same.

Dr. Ulrich, of Pennsylvania, said, in regard to the inflammatory origin of consumption, that he had lived for twenty years on the banks of the Mississippi, in Louisiana, and had never known a case of tuberculosis to originate in that locality, and yet the people suffered a great deal from pneumonia. He then reiterated what he said in a former discussion, that consumption is caused by the wretched manner in which some people live, and their in-door employment.

